# Interactions between Diet, Lifestyle and *IL10, IL1B*, and *PTGS2/COX-2* Gene Polymorphisms in Relation to Risk of Colorectal Cancer in a Prospective Danish Case-Cohort Study

**DOI:** 10.1371/journal.pone.0078366

**Published:** 2013-10-23

**Authors:** Vibeke Andersen, René Holst, Tine Iskov Kopp, Anne Tjønneland, Ulla Vogel

**Affiliations:** 1 Organ Center, Hospital of Southern Jutland, Aabenraa, Denmark; 2 Institute of Regional Health Research, University of Southern Denmark, Odense, Denmark; 3 Medical Department, Regional Hospital, Viborg, Viborg, Denmark; 4 National Food Institute, Soborg, Denmark; 5 Danish Cancer Society Research Center, Copenhagen, Denmark; 6 National Research Centre for the Working Environment, Copenhagen, Denmark; MOE Key Laboratory of Environment and Health, School of Public Health, Tongji Medical College, Huazhong University of Science and Technology, China

## Abstract

**Background & Aims:**

Diet contributes to colorectal cancer development and may be potentially modified. We wanted to identify the biological mechanisms underlying colorectal carcinogenesis by assessment of diet-gene interactions.

**Methods:**

The polymorphisms *IL10* C-592A (rs1800872), C-rs3024505-T, *IL1b* C-3737T (rs4848306), G-1464C (rs1143623), T-31C (rs1143627) and *PTGS2* (encoding COX-2) A-1195G (rs689466), G-765C (rs20417), and T8473C (rs5275) were assessed in relation to risk of colorectal cancer (CRC) and interaction with diet (red meat, fish, fibre, cereals, fruit and vegetables) and lifestyle (non-steroid-anti-inflammatory drug use and smoking status) was assessed in a nested case-cohort study of nine hundred and seventy CRC cases and 1789 randomly selected participants from a prospective study of 57,053 persons.

**Results:**

*IL1b* C-3737T, G-1464C and *PTGS2* T8473C variant genotypes were associated with risk of CRC compared to the homozygous wildtype genotype (IRR=0.81, 95%CI: 0.68-0.97, p=0.02, and IRR=1.22, 95%CI: 1.04-1.44, p=0.02, IRR=0.75, 95%CI: 0.57-0.99, p=0.04, respectively). Interactions were found between diet and *IL10* rs3024505 (P-value for interaction (P_int_); meat=0.04, fish=0.007, fibre=0.0008, vegetables=0.0005), C-592A (P_int_; fibre=0.025), *IL1b* C-3737T (P_int_; vegetables=0.030, NSAID use=0.040) and *PTGS2* genotypes G-765C (P_int_; meat=0.006, fibre=0.0003, fruit 0.004), and T8473C (P_int_; meat 0.049, fruit=0.03) and A-1195G (P_int_; meat 0.038, fibre 0.040, fruit=0.059, vegetables=0.025, and current smoking=0.046).

**Conclusions:**

Genetically determined low COX-2 and high IL-1β activity were associated with increased risk of CRC in this northern Caucasian cohort. Furthermore, interactions were found between *IL10*, *IL1b*, and *PTGS2* and diet and lifestyle factors in relation to CRC. The present study demonstrates that gene-environment interactions may identify genes and environmental factors involved in colorectal carcinogenesis.

## Introduction

Colorectal cancer (CRC) is one of the most common cancers in the Western World [1]. Increasing incidence suggests that lifestyle factors are deeply involved in the etiology of CRC and, that modification of these factors may affect risk [2]. The assessment of gene-environment interactions provides a tool for understanding the underlying biological pathways by which diet affects colorectal carcinogenesis [3–5]. This topic has recently been reviewed [6].

Chronic intestinal inflammation is a well-known risk factor for CRC [7]. Diet and lifestyle factors may affect intestinal inflammation in many ways, directly or indirectly. Meat, for example, has been found to affect the intestinal homeostasis e.g. by activation of pattern recognition receptors such as toll-like receptors (TLRs) [8]. Also, meat is a source of n-6 poly-unsaturated fatty acids (PUFA) which may undergo metabolic conversion to arachidonic acid and predominantly pro-inflammatory prostaglandins [9]. Fish is a source of n-3 PUFA, which may modify inflammation [10]. Furthermore, dietary fibre from vegetables, fruit and cereals are converted by colonic bacteria to short-chain fatty acids (SCFA) which have been found to affect intestinal inflammation in various ways including stimulation of IL-10 production [11]. 

IL-10, IL-1β and COX-2 (encoded by *IL10*, *IL1B*, and *PTGS2*, respectively), are important mediators of intestinal inflammation. Both SCFA and TLR activation have been found to affect production of IL-10 and IL-1β and thereby COX-2 activation [11,12]. IL-10 is a key anti-inflammatory cytokine orchestrating the innate and adaptive immune response. IL-10 -/- mice develop colitis and subsequently colorectal adenocarcinomas [13]. IL-1β is a proinflammatory cytokine and genetic variation in *IL1B* has been associated with risk of lung cancer and multiple myeloma [14,15]. A central function of COX-2 in colorectal carcinogenesis is suggested by the finding that long term use of COX-2 inhibitors (COXIB) has been found to confer protection against CRC in some studies [16]. 

The use of functional polymorphisms has the advantage that the results may allow interpretation of the involved biological pathways in colorectal carcinogenesis. 

We have previously assessed diet and *IL10* gene interactions in a prospective Danish cohort of three hundred and seventy-eight CRC cases and a comparison group of 775 participants [17]. We found no association with CRC *per see*, yet, we found interactions between *IL10* polymorphisms and intake of dietary fibre [17]. We have also previously assessed genetic variation in *IL1B* and *PTGS2* in this cohort, finding no statistically significant associations with risk of CRC [3,18]. We now extend our studies to a larger cohort with more than twice the number of cases and members of the comparison group and include more dietary factors and all the three functional promoter polymorphisms in *IL1B*.

Therefore, we assessed the functional polymorphisms *IL10* C-592A (rs1800872), *IL1B* C-3737T (rs4848306), G-1464C (rs1143623), T-31C (rs1143627) and *PTGS2* (encoding COX-2) A-1195G (rs689466), G-765C (rs20417), T8473C (rs5275) and the *IL10* marker polymorphism C-rs3024505-T in relation to diet (red meat, fish, fibre, cereals, fruit and vegetables) and lifestyle (non-steroid-anti-inflammatory drug use and smoking status) in a nested case-cohort study of nine hundred and seventy CRC cases and 1789 randomly selected participants from the prospective Diet, Cancer and Health study encompassing 57,053 persons. 

## Methods

### Studied Subjects

The Diet, Cancer and Health Study is an ongoing Danish cohort study designed to investigate the relation between diet, lifestyle and cancer risk [19]. The cohort consists of 57,053 persons, recruited between December 1993 and May 1997. All the subjects were born in Denmark, and the individuals were 50 to 64 years of age and had no previous cancers at study entry. Blood samples and questionnaire data on diet and lifestyle were collected at study entry. 

### Follow-up and endpoints

Follow-up was based on population-based cancer registries. Between 1994 and 31th December 2009, nine hundred and seventy CRC cases were diagnosed. A subcohort of 1897 persons was randomly selected within the cohort. Of these, 108 with missing genotype data were excluded. All information on genotypes and diet and lifestyle factors was available for nine hundred and seventy CRC cases and 1789 subcohort members. 

### Dietary and lifestyle questionnaire

Information on diet, lifestyle, weight, height, medical treatment, environmental exposures, and other socio-economic factors were collected at enrolment using questionnaires and interviews. In the food-frequency questionnaire, diet consumption was assessed in 12 categories of predefined responses, ranking from ’never’ to ’eight times or more per day’. The daily intake was then calculated by using FoodCalc [19]; this program uses population specific standardized recipes and portion sizes. Intake of red meat in grams per day was calculated by adding up intake of beef, veal, pork, lamb and offal. Intake of processed meat in grams per day was calculated by adding up intake of processed red meat, including bacon, smoked ham, salami, frankfurter, Cumberland sausage, cold cuts and liver pâté. Dietary fibre intake was based on country-specific food composition tables, which were reviewed to ensure comparability to the association of official analytical chemists (AOAC) fibre definition, which includes lignin and resistant starch [20]. Fibre intake is calculated by multiplying the frequency of consumption of relevant foods by their fibre content as determined from national databases of food content [21]. 

Contributing food items to the food group ‘cereals’ included wholegrain foods (wholegrain bread, rye bread, wholegrain flour, oatmeal, corncobs, müsli, and crispbread) and refined grain foods (white wheat bread, wheat flour, rice flour, potato flour, corn flour/starch, pasta, wheat) and was measured in grams per day [22]. Intake of ‘fish’ in grams per day was calculated by adding up intake of fresh and processed fish. For fruit, only intake of fresh fruit (as indicated on the FFQ) was examined, while vegetable intake also included estimated contributions from recipes. The questionnaire was tested in a validation study preceding the Diet, Cancer and Health study. Pearson correlation coefficients (adjusted for total energy intake) illustrating the comparison of nutrient scores estimated from the food-frequency questionnaire and from weighed diet records were 0.39 and 0.53 for dietary fibre and 0.37 and 0.14 for meat for men and women, respectively [23,24].

Smoking status was classified as never, past or current. Persons smoking at least 1 cigarette daily during the last year were classified as smokers.

The lifestyle questionnaire included this question regarding use of NSAID: “Have you taken more than one pain relieving pill per month during the last year?” If the answer was yes, the participant was asked to record how frequently they took each of the following medications: “Aspirin”, “Paracetamol”, “Ibuprofen”, or “Other pain relievers”. The latter category included NSAID preparations other than aspirin and ibuprofen. Based on all records, we classified study subjects according to use of “any NSAID” (≥ 2 pills per month during one year) at baseline. 

### Genotyping

Buffy coat preparations were stored at minus 150°C until use. DNA was extracted as described [25]. The DNA was genotyped by KBioscience (KBioscience, Hoddesdon, United Kingdom) by PCR-based KASP™ genotyping assay. (http://www.lgcgenomics.com/). One marker polymorphism, the *IL10* C-rs3024505-T, and 8 functional polymorphisms were selected; *IL10* C-592A (rs1800872), *IL1B* C-3737T (rs4848306), G-1464C (rs1143623), T-31C (rs1143627) and *PTGS2* (encoding COX-2) A-1195G (rs689466), G-765C (rs20417), and T8473C (rs5275). *IL1B* T-31C (rs1143627) [18], *PTGS2* (encoding COX-2) A-1195G (rs689466), G-765C (rs20417), and T8473C (rs5275) [3] were determined and reported previously for a subset of the study group. Furthermore, all the three SNPs in IL1B were determined for the whole comparison group independently of the present work and reported previously [26]. To confirm reproducibility, genotyping was repeated for 10 % of the samples yielding 100% identity. 

### Statistical Analysis

Deviation from Hardy-Weinberg equilibrium was assessed using a Chi square test.

Incidence rate ratios (IRR) and 95% Confidence Interval (95%CI) were calculated according to the principles for analysis of case-cohort studies using an un-weighted approach [27]. Age was used as the time scale in the Cox regression models. Tests and confidence intervals were based on Wald’s tests using the robust estimate of the variance-covariance matrix for the regression parameters in the Cox regression models [28] as previously described [3,5,17,18,29–34]. 

All models were adjusted for baseline values of suspected risk factors for colorectal cancer such as body mass index (BMI) (kg/m^2^, continuous), NSAID (yes/no), use of hormone replacement therapy (HRT) (never/past/current, among women), smoking status (never/past/current), intake of dietary fibre (g/day, continuous), and red meat and processed meat (g/day, continuous). Cereals, fibre, fruit and vegetables were also entered linearly. All analyses were stratified by gender, so that the basic (underlying) hazards were gender specific. For all the polymorphisms, IRR was calculated separately for heterozygous and homozygous variant allele carriers. For all the SNPs except for *PTGS2* A-1195G, all variant allele carriers were subsequently grouped for interaction analyses since no recessive effects were observed. For *PTGS2* A-1195G, a recessive mode was used in the subsequent analyses.

Haplotypes of *PTGS2* and *IL1B* were inferred manually as done previously [3,35,36].

For the different genes, we investigated possible interactions between the polymorphisms and intake of meat, dietary fibre, cereals, fish, fruit and vegetables, smoking status and NSAID use using the likelihood ratio test [3,14,32,35–37]. 

In another set of interaction analyses between the polymorphisms and the dietary intake subdivided in tertiles, dietary intake was entered as a categorical variable. Tertile cut-points were based on the empirical distribution among cases. The possible interactions were investigated using the likelihood ratio test. 

All analyses were performed using R version 2.15-1 (R Core Team, 2013) [38]. A p<0.05 was considered to be significant. 

### Ethics Statement

All participants gave verbal and written informed consent. The Diet, Cancer and Health study was approved by the National Committee on Health Research Ethics (journal nr. (KF) 01-345/93) and the Danish Data Protection Agency.

## Results

Characteristics of the study population and risk factors for CRC are shown in Table 1. The genotype distribution of the polymorphisms in the sub-cohort did not deviate from Hardy-Weinberg equilibrium (results not shown). The variant allele frequency in the sub-cohort were for *IL10* C-592A 0.22, rs3024505 0.17, *IL1B* C-3737T 0.43, G-1464C 0.27, T-31C 0.33 and *PTGS2* A-1195G 0.19, G-765C 0.15, and T8473C 0.34, respectively.

**Table 1:** Baseline characteristics of study population selected for the Diet, Cancer and Health cohort.

	Cases			Sub-cohort		Test for difference
	No.	Medians		No.	Medians	p-value
	(%)	(5-95% percentiles)		(%)	(5-95% percentiles)	
Total	970 (100)			1789(100)		
Sex						0.13
Men	547(56)			954 (53)		
Women	423(44)			835 (47)		
Age at inclusion (*years*)		58 (51-64)			56 (50-64)	<1e-16
BMI ( *kg/m* ^*2*^)		26.3 (20.7-34.3)			25.6 (20.5-33.0)	0.001
Food intake (*g/day*)						
Alcohol^1^		14.0 (0.5-69.9)			13.5 (0.7-65.4)	0.23
Dietary fiber		20.0 (10.6-32.8)			20.6 (10.8-34.2)	0.01
Red and processed meat		113 (47-233)			109 (42-236)	0.03
Smoking status						0.07
Never	286 (30)			603 (34)		
Past	301 (31)			518 (29)		
Current	383 (40)			667 (37)		
NSAID use						0.65
No	699 (70)			1218 (69)		
Yes	293 (30)			557 (31)		
HRT use among women^2^						0.01
Never	258 (61)			437 (52)		
Past	55 (13)			132 (16)		
Current	110 (26)			266 (32)		

^1^ Among current drinkers

^2^ Percentages among female cases/members of the comparison group

### Associations between polymorphisms and CRC

*IL1B* C-3737T variant allele carriers were at lowered risk of CRC and the G-1464C variant allele carriers were at higher risk of CRC compared to the homozygous wildtype genotype carriers (p=0.02 and p=0.02, respectively) (Table 2). Haplotype analyses revealed that the *IL1B* haplotype combinations which included the CCC haplotype (C-3737T, G-1464C, T-31C) were associated with increased risk of CRC compared to the reference TGT/TGT haplotype (Table S1). However, only the haplotype combination CCC/CGT were statistically significantly associated with risk of CRC (p=0.02). Carriers of one copy of the haplotype CCC had an IRR of 1.20 (p=0.04) and carriers of two CCC haplotypes had an IRR of 1.29 (p=0.12) (reference group: no CCC haplotype) (Table 3). Carriers of one copy of the TGT haplotype had an IRR of 0.82 (p=0.04) and carriers of two TGT copies had an IRR of 0.79 (p=0.05) (Table 3).

**Table 2:** Incidence rate ratios and 95% confidence intervals for the studied gene polymorphisms in the Diet, Cancer and Health study.

		N_case_	N_sub-cohort_	Crude^a^			Adjusted^b^			P-value^c^
				IRR	(95%CI)		IRR	(95%CI)		
***IL10*** C-592A										
	CC	596	1072	1.00			1.00			
	AC	297	580	0.92	(0.78-1.10)		0.92	(0.77-1.10)		0.38
	AA	56	96	1.02	(0.71-1.45)		1.00	(0.70-1.44)		0.98
	AC-AA	353	676	0.94	(0.79-1.11)		0.93	(0.79-1.11)		0.44
***IL10*** rs3024505										
	CC	648	1200	1.00			1.00			
	CT	263	511	0.97	(0.81-1.16)		0.98	(0.82-1.18)		0.87
	TT	34	54	1.03	(0.66-1.63)		0.99	(0.62-1.58)		0.96
	CT-TT	297	565	0.98	(0.82-1.16)		0.98	(0.83-1.17)		0.87
***IL1B*** C-3737T										
	CC	336	560	1.00			1.00			
	CT	433	835	0.84	(0.70-1.01)		0.82	(0.68-0.99)		0.04
	TT	172	351	0.79	(0.63-1.00)		0.79	(0.63-1.01)		0.06
	CT-TT	605	1186	0.83	(0.70-0.98)		0.81	(0.68-0.97)		0.02
***IL1B*** G-1464C										
	GG	454	925	1.00			1.00			
	CG	408	683	1.21	(1.02-1.43)		1.21	(1.02-1.44)		0.03
	CC	84	141	1.26	(0.93-1.71)		1.30	(0.95-1.77)		0.10
	CG-CC	492	824	1.21	(1.03-1.43)		1.22	(1.04-1.44)		0.02
***IL1B*** T-31C										
	TT	389	773	1.00			1.00			
	TC	440	779	1.10	(0.93-1.31)		1.11	(0.93-1.32)		0.26
	CC	117	204	1.22	(0.94-1.59)		1.22	(0.93-1.59)		0.16
	TC-CC	557	983	1.13	(0.96-1.33)		1.13	(0.95-1.33)		0.16
***PTGS2*** A-1195G										
	AA	587	1126	1.00			1.00			
	AG	313	560	1.06	(0.89-1.27)		1.07	(0.90-1.28)		0.43
	GG	47	61	1.41	(0.94-2.11)		1.46	(0.97-2.20)		0.07
	AA-AG vs GG^d^	900	1686	1.38	(0.93-2.05)		1.42	(0.95-2.14)		0.09
***PTGS2*** G-765C										
	GG	701	1256	1.00			1.00			
	GC	213	435	0.90	(0.74-1.09)		0.86	(0.71-1.05)		0.14
	CC	22	43	0.91	(0.54-1.54)		0.96	(0.56-1.63)		0.88
	GC-CC	235	478	0.90	(0.75-1.08)		0.87	(0.72-1.05)		0.15
***PTGS2*** T8473C										
	TT	430	720	1.00			1.00			
	CT	404	815	0.86	(0.72-1.02)		0.84	(0.71-1.01)		0.06
	CC	97	203	0.77	(0.59-1.02)		0.75	(0.57-0.99)		0.04
	CT-CC	501	1018	0.84	(0.71-0.99)		0.82	(0.70-0.97)		0.02

^a^ Adjusted for sex and age

^b^ In addition, adjusted for smoking status, alcohol, HRT status (women only), BMI, use of NSAID, and intake of red and processed meat, and dietary fibre

^c^ P-value for the adjusted estimates

^d^ AA and AG versus GG.

**Table 3:** Risk estimates for *IL1B* and *PTGS2* haplotypes in relation to risk of colorectal cancer.

		**Copy**	**N_cases_**	**N_subcohort_**	**IRR^a^**	**(95%CI)**		**IRR^b^**	**(95%CI)**		**P-value^c^**
*IL1B*	TGT	0	330	563	1			1			
		1	424	840	0.84	(0.70-1.01)		0.82	(0.68-0.99)		0.035
		2	168	353	0.78	(0.62-0.99)		0.79	(0.62-1.00)		0.051
	CCC	0	444	929	1			1			
		1	397	688	1.20	1.02-1.43)		1.20	(1.01-1.43)		0.040
		2	81	139	1.25	(0.92-1.70)		1.29	(0.94-1.76)		0.116
*PTGS2*	GGT	0	560	1104	1			1			
		1	296	559	1.05	(0.88-1.25)		1.06	(0.89-1.27)		0.514
		2	46	57	1.58	(1.04-2.38)		1.62	(1.06-2.47)		0.024
	AGT	0	144	267	1			1			
		1	573	1110	1.01	(0.80-1.27)		1.01	(0.80-1.27)		0.952
		2	185	343	1.05	(0.80-1.39)		1.05	(0.79-1.39)		0.750

Haplotype sequence: *IL1B*: C-3737T, G-1464C, T-31C. PTGS2: A-1195G, G-765C, T8473C

^a^ Adjusted for sex and age

^b^ In addition, adjusted for smoking status, alcohol, HRT status (women only), BMI, intake of red and processed meat, and dietary fibre

^c^ P-value for the adjusted risk estimates

Carriers of the high COX-2 activity *PTGS2* T8473C variant allele were at lower risk of CRC and homozygous carriers of the low COX-2 activity *PTGS2* A-1195G variant G-allele were at marginally higher risk of CRC than the homozygous wildtype genotype (p=0.02 and p=0.07, respectively) (Table 2). Furthermore, carriers of the haplotype combination which included both copies of the A-1195G variant alleles (GGT/GGT), were at increased risk of CRC (p=0.09) compared to the reference *PTGS2* AGT/AGT (A-1195G, G-765C, T8473C) haplotype combination (Table S1). In a separate analysis, carriers of one GGT copy had an IRR of 1.06 (p=0.51) whereas carriers of two copies had an IRR of 1.62 compared to all non-carriers of the haplotype (p=0.02) (Table 3). 

Where no recessive effects were observed, variant genotypes were combined in the interaction analysis to maximize the statistical power. A recessive effect was found for *PTGS2* A-1195G and consequently, AA and AG carriers were grouped versus GG carriers. 

### Gene-environment analyses

#### Meat

*IL10* rs3024505 variant carriers were at 6% increased risk of CRC per 25 g red and processed meat per day (95%CI: 1.00-1.11) whereas homozygous wildtype carriers were at no risk by meat intake (P-value for interaction (P_int_)=0.04) (Table 4). These findings were supported by the tertile analyses (Table S2). *IL10* rs3024505 variant carriers were at 1.50 increased risk by high meat intake compared to homozygous wildtype carriers with low meat intake (95%CI: 1.09-2.06, P_int_=0.02).

**Table 4:** Interaction between dietary factors and the studied polymorphisms in relation to colorectal cancer risk.

			**IRR^a^**	**(95%CI)**	**IRR^b^**	**(95%CI)**	**P-value**		**IRR^a^**	**(95%CI)**	**IRR^b^**	**(95%CI)**	**P-value**		**IRR^a^**	**(95%CI)**	**IRR^b^**	**(95%CI)**	**P-value^c^**
			***Red and processed meat per 25 g/day***						***Fish per 25 g/day***						***Dietary cereal per 50 g/day***				
***IL10***	C-592A	CC	1.03	(0.99-1.07)	1.02	(0.98-1.06)			0.94	(0.85-1.04)	0.94	(0.85-1.04)			0.95	(0.89-1.02)	0.97	(0.90-1.04)	
		AC-AA	1.01	(0.96-1.07)	1.00	(0.95-1.06)	0.4553		0.99	(0.88-1.12)	0.98	(0.86-1.11)	0.5596		0.93	(0.85-1.02)	0.95	(0.86-1.04)	0.6419
	rs3024505	CC	1.01	(0.97-1.05)	1.00	(0.96-1.04)			0.91	(0.83-1.00)	0.90	(0.82-0.99)			0.93	(0.86-0.99)	0.94	(0.87-1.02)	
		CT-TT	1.06	(1.01-1.11)	1.06	(1.00-1.11)	0.0361		1.08	(0.94-1.24)	1.08	(0.94-1.24)	0.0065		0.98	(0.90-1.07)	0.99	(0.90-1.09)	0.2715
***IL1B***	C-3737T	CC	1.01	(0.96-1.07)	1.01	(0.96-1.07)			0.98	(0.86-1.11)	0.98	(0.86-1.11)			0.98	(0.89-1.08)	1.00	(0.90-1.11)	
		CT-TT	1.03	(0.99-1.07)	1.02	(0.98-1.06)	0.6519		0.94	(0.86-1.04)	0.93	(0.84-1.03)	0.4590		0.92	(0.86-0.99)	0.94	(0.87-1.01)	0.1743
	G-1464C	GG	1.02	(0.98-1.07)	1.02	(0.97-1.06)			0.95	(0.86-1.05)	0.94	(0.85-1.04)			0.93	(0.86-1.01)	0.95	(0.88-1.03)	
		GC-CC	1.03	(0.98-1.08)	1.02	(0.97-1.07)	0.9367		0.97	(0.86-1.08)	0.97	(0.86-1.09)	0.6227		0.95	(0.88-1.03)	0.96	(0.88-1.05)	0.7630
	T-31C	TT	1.01	(0.96-1.05)	1.00	(0.96-1.05)			0.93	(0.83-1.03)	0.91	(0.81-1.03)			0.92	(0.85-1.00)	0.95	(0.87-1.03)	
		TC-CC	1.04	(0.99-1.08)	1.03	(0.98-1.07)	0.3954		0.98	(0.88-1.08)	0.98	(0.88-1.09)	0.3089		0.95	(0.88-1.03)	0.96	(0.89-1.04)	0.7252
***PTGS2***	A-1195G	AA-AG	1.02	(0.99-1.06)	1.02	(0.98-1.05)			0.96	(0.89-1.04)	0.96	(0.8481.04)			0.95	(0.90-1.01)	0.97	(0.91-1.03)	
		GG	1.05	(0.87-1.27)	1.06	(0.87-1.29)	0.5439		0.82	(0.54-1.23)	0.78	(0.51-1.19)	0.2116		0.77	(0.58-1.03)	0.77	(0.58-1.04)	0.0387
	G-765C	GG	1.00	(0.96-1.04)	0.99	(0.95-1.03)			0.93	(0.85-1.01)	0.92	(0.84-1.01)			0.93	(0.87-0.99)	0.94	(0.88-1.01)	
		GC-CC	1.08	(1.01-1.15)	1.08	(1.01-1.15)	0.0058		1.05	(0.90-1.23)	1.05	(0.89-1.25)	0.0663		0.99	(0.89-1.10)	1.01	(0.91-1.12)	0.1771
	T8473C	TT	1.04	(0.99-1.10)	1.04	(0.99-1.09)			0.95	(0.85-1.05)	0.94	(0.84-1.05)			0.93	(0.86-1.01)	0.95	(0.87-1.03)	
		TC-CC	1.01	(0.97-1.06)	1.01	(0.96-1.05)	0.2924		0.96	(0.87-1.07)	0.95	(0.85-1.07)	0.8029		0.94	(0.87-1.02)	0.96	(0.88-1.04)	0.8573
			***Dietary fibre per 10 g/day***						***Fruit per 50 g/day***						***Vegetables per 50 g/day***				
***IL10***	C-592A	CC	0.87	(0.76-1.00)	0.91	(0.78-1.05)			0.97	(0.94-1.00)	0.98	(0.94-1.01)			0.98	(0.93-1.03)	0.99	(0.94-1.04)	
		AC-AA	0.79	(0.64-0.96)	0.79	(0.64-0.98)	0.1638		0.96	(0.92-1.00)	0.96	(0.92-1.00)	0.3443		0.96	(0.89-1.04)	0.97	(0.89-1.04)	0.5043
	rs3024505	CC	0.75	(0.65-0.87)	0.77	(0.66-0.89)			0.96	(0.93-0.99)	0.96	(0.93-0.99)			0.93	(0.89-0.99)	0.94	(0.89-0.99)	
		CT-TT	1.03	(0.85-1.25)	1.06	(0.87-1.30)	0.0008		0.98	(0.93-1.03)	0.99	(0.94-1.04)	0.1891		1.04	(0.98-1.11)	1.06	(0.99-1.13)	0.0005
***IL1B***	C-3737T	CC	0.81	(0.66-0.99)	0.83	(0.67-1.02)			0.96	(0.93-1.00)	0.96	(0.92-1.00)			0.94	(0.87-1.01)	0.94	(0.86-1.02)	
		CT-TT	0.85	(0.73-0.98)	0.88	(0.75-1.02)	0.5695		0.96	(0.93-1.00)	0.97	(0.94-1.00)	0.7166		0.98	(0.94-1.03)	1.00	(0.95-1.05)	0.0705
	G-1464C	GG	0.83	(0.71-0.98)	0.87	(0.74-1.03)			0.97	(0.93-1.00)	0.97	(0.94-1.01)			0.95	(0.90-1.00)	0.96	(0.91-1.02)	
		GC-CC	0.84	(0.71-0.99)	0.85	(0.72-1.01)	0.8181		0.96	(0.92-1.00)	0.96	(0.92-1.00)	0.5955		1.00	(0.93-1.06)	1.00	(0.94-1.07)	0.2082
	T-31C	TT	0.82	(0.69-0.97)	0.86	(0.72-1.03)			0.97	(0.93-1.01)	0.98	(0.94-1.01)			0.96	(0.91-1.02)	0.97	(0.92-1.03)	
		TC-CC	0.85	(0.73-0.99)	0.86	(0.73-1.01)	0.9904		0.96	(0.92-0.99)	0.96	(0.93-1.00)	0.4715		0.97	(0.92-1.03)	0.98	(0.92-1.05)	0.8110
***PTGS2***	A-1195G	AA-AG	0.85	(0.76-0.96)	0.88	(0.78-1.00)			0.96	(0.94-0.99)	0.97	(0.94-0.99)			0.97	(0.93-1.01)	0.98	(0.94-1.03)	
		GG	0.49	(0.23-1.07)	0.47	(0.21-1.05)	0.0224		0.94	(0.79-1.12)	0.94	(0.77-1.12)	0.6246		0.92	(0.77-1.11)	0.91	(0.77-1.09)	0.3053
	G-765C	GG	0.76	(0.67-0.87)	0.79	(0.68-0.91)			0.95	(0.92-0.98)	0.95	(0.92-0.98)			0.96	(0.92-1.01)	0.98	(0.93-1.03)	
		GC-CC	1.13	(0.90-1.40)	1.16	(0.93-1.46)	0.0003		1.02	(0.96-1.07)	1.02	(0.97-1.07)	0.0041		1.01	(0.93-1.10)	1.02	(0.94-1.12)	0.2457
	T8473C	TT	0.76	(0.63-0.90)	0.79	(0.65-0.94)			0.94	(0.90-0.97)	0.94	(0.91-0.98)			0.96	(0.90-1.02)	0.97	(0.91-1.03)	
		TC-CC	0.92	(0.79-1.07)	0.94	(0.80-1.10)	0.0684		0.99	(0.95-1.02)	0.99	(0.96-1.02)	0.0333		0.99	(0.93-1.05)	1.00	(0.94-1.06)	0.3800

^a^ Crude adjusted for sex and age

^b^ Adjusted for smoking status, Alcohol, HRT status (women only), BMI, intake of red and processed meat, and dietary fibre

^c^ P p-value for interaction the adjusted risk estimates

*PTGS2* G-765C variant allele carriers were at 8% increased risk of CRC per 25 g red and processed meat per day (95% CI: 1.00-1.15) whereas homozygous wildtype carriers were at no risk by meat intake (P_int_=0.006) (Table 4). Also, *PTGS2* G-765C variant allele carriers were at increased risk of CRC by meat intake in the tertile analysis compared to the homozygous wildtype carriers (P_int_=0.005) (Table S2). 

#### Fish

*IL10* rs3024505 homozygous wildtype carriers were at 10 % reduced risk of CRC per 25 g fish per day whereas variant carriers had no risk reduction by similar intake (P_int_=0.007).

#### Fibre, fruit, vegetables, and cereals

*IL10* rs3024505 homozygous wildtype carriers were at 23 and 6 % reduced risk of CRC per 10 g fibre and 50 g vegetables per day whereas variant carriers had no risk reduction by similar intake (P_int_=0.0008, and 0.0005, respectively). Furthermore, *IL10* rs3024505 homozygous wildtype carriers were at lowered risk of CRC among study participants with the highest intake of fibre (IRR=0.73, 95%CI: 0.57-0.94, P_int_=0.007) and vegetables (IRR=0.72, 95%CI: 0.56-0.93, P_int_=0.001). 

*PTGS2* G-765C homozygous wildtype carriers were at 21% and 5% reduced risk of CRC per 10 g fibre and 50 g fruit per day whereas variant carriers had no risk reduction by similar intake (P_int_=0.0003, and 0.004, respectively). In the tertile analysis, *PTGS2* G-765C homozygous wildtype carriers were at low risk of CRC by high intake of fibre (IRR=0.71, 95% CI: 0.55-0.90, P_int_=0.004), and fruit (IRR=0.73, 95% CI: 0.57-0.93, P_int_=0.006).No interaction between any genotypes and cereal in relation to risk of CRC was found (Table 4). In the tertile analyses, *IL1B* G-3737C variant allele carriers were at lowered risk of CRC in the lowest tertile of vegetables (IRR=0.66, 95% CI: 0.49-0.89) whereas the risk estimates in the highest tertile was similar for the two alleles (P_int_=0.03) (Table S2). 

#### NSAID use

A statistically significant association between *IL1B* C-3737T and use of NSAID was found (Table S3). Low risk of CRC was found for the *IL1B* C-3737T variant allele carriers among non-NSAID users (IRR=0.74, 95% CI: 0.60-0.91) but not among NSAID users (IRR=0.82, 95% CI: 0.64-1.06) compared to the homozygous wildtype carriers (reference) (P_int_=0.04).

#### Smoking

A statistically significant association between *PTGS2* A-1195G and smoking was found (Table S4). Among current smokers, homozygous *PTGS2* A-1195G variant allele carriers were at higher risk of CRC (IRR=2.33, 95% CI: 1.13-4.78, P_int_=0.046) compared to homozygous wildtype carriers who had never smoked (reference group). 

## Discussion

In the present candidate gene study, we analysed gene-environment interactions in relation to risk of CRC in a Danish prospective cohort. We found that functional *IL1B* and *PTGS2* polymorphisms were associated with risk of CRC (Table 2 and 3, and Table S1). Furthermore, we found interactions between diet and lifestyle factors and genes involved in the inflammatory pathway (Table 3 and Table S2, S3 and S4). Thus, we found interactions between intake of meat and *IL10* and *PTGS2*, fish and *IL10*, fibre and *IL10* and *PTGS2*, fruit and *PTGS2*, vegetables and *IL10*, *PTGS2*, and *IL1B*, NSAID use and *IL1B*, and, finally, between smoking status and *PTGS2* polymorphisms. 

### Associations between polymorphisms and CRC

We now extend our previous studies of *IL10*, *IL1B* and *PTGS2* polymorphisms in relation to diet and colorectal carcinogenesis in a study group of three hundred and seventy-eight CRC cases and 775 participants in a randomly selected comparison group [3,17,18] studies to a larger cohort with more than twice the number of cases and members of the comparison group and include more dietary factors. In contrast to our previous findings [3,18], the studied functional polymorphisms in *IL1B* and *PTGS2* were associated with risk of CRC in the present larger study group probably reflecting the increased statistical power. We reproduced the previous findings that the studied IL10 polymorphisms were not associated with risk of CRC *per* see 17.

In the present study, the *IL1B* CCC (C-3737T, G-1464C, T-31C) haplotype was associated with increased risk of CRC. This haplotype gives high transcription levels in studies of the promoter using transient transfections [39]. Conversely, the TGT haplotype was associated with low CRC risk. This haplotype leads to low *IL1B* transcription and encompasses the variant allele of C-3737T. The *IL1B* C-3737T polymorphism abolishes a binding site for the anti-inflammatory NF-κB subunit p50 [39]. Thus, our results, both in terms of SNP analyses and haplotype analyses consistently indicate that genetically determined high IL1B levels are associated with increased risk of CRC and genetically low IL1B levels leads to lowered risk of CRC. Furthermore, our result may suggest involvement of the anti-inflammatory p50 subunit of NF-κB. We found no statistically significant interaction between the studied *IL1B* polymorphisms and any of the studied diet variables. However, in the tertile analysis of vegetable intake (table S2) the association between the *IL1B* polymorphisms and risk of CRC was strongest in the lowest tertile. Thus, carriers of the variant allele of *IL1B* G-1464C and T-31C were at 1.40 (95%CI: 1.05-1.86) and 1.47 (95%CI: 1.11-1.95) fold increased risk of CRC, respectively, whereas carriers of the variant allele of C-3737T were at reduced risk of CRC (IRR=0.66, 95% CI: 0.49-0.89). This may suggest that the association between IL1B polymorphisms and risk of CRC can only be detected in populations with relatively low vegetable intake such as the Danish population [40]. 

The *PTGS2* A-1195G variant allele leads to low transcription levels of COX-2 [41] whereas *PTGS2* T8473C gives high mRNA levels [42]. In Danes including the present study group, the variant allele of G-765C almost exclusively co-segregates with the variant allele of T8473C (Table S1). This haplotype has been shown to be associated with highly elevated COX-2 activity [43]. In the present study, the *PTGS2* GGT (A-1195G, G-765C, T8473C) haplotype was associated with increased risk of CRC (P=0.024). The *PTGS2* A-1195G homozygous variant genotype was marginally associated with increased risk of CRC (P=0.07) and *PTGS2* T8473C variant carriers with genetically determined high COX-2 activity were at lowered risk of CRC (P=0.02). In accordance with the present study, genetically low COX-2 activity was found to predispose to inflammatory bowel disease, a risk factor for CRC [44].

### Gene-environment analyses

The intake of meat in the Danish population is among the highest intakes world-wide and we have previously identified interactions between meat and genes [3,5]. We found interaction between intake of meat and *PTGS2* G-765C. Thus, among variant allele carriers, daily intake of meat was associated with 8% increased risk of CRC pr 25g meat, whereas homozygous wildtype allele carriers were not at increased risk (P_int_=0.006). The result is in accordance with the finding of a statistically significant association between *PTGS2* G-765C variant genotypes and CRC among subjects with high n-6 PUFA intake in a prospective, population-based cohort of 310 Singapore Chinese cases [45]. N-6 PUFA is present in meat. We also found interaction between meat intake and *IL10* rs3024505. A similar interaction was found for fish intake. However, it is difficult to interpret the functional implications as *IL10* rs3024505 is a marker SNP with no known function. The lack of interaction with the functional promoter polymorphism C-592A may suggest that the detected interaction may be related to other genes than *IL10*, but on the other hand, *IL10* rs3024505 is located very far away from other genes [46]. In summary, the interactions between meat intake on one hand and genetic variation in *PTGS2* and *IL10* on the other hand, suggest that inflammation plays a role in meat related carcinogenesis. In support of this, we have also found interaction between the functional promoter polymorphism *NFKB1* -94ins/del and meat intake in relation to CRC [5].

We observed strong interaction between the marker *IL10* rs3024505 and intake of fibre and vegetables. In both cases, homozygous wildtype allele carriers benefited from high intake, whereas variant allele carriers had no risk reduction when eating fibre or vegetables in relation to CRC risk. However, since variant allele carriers in the tertile with the lowest fibre intake were at marginally lowered risk of CRC (IRR: 0.73, 95% CI: 0.54-1.01) the results suggest that wildtype allele carriers experience a risk reduction by fibre intake that variant allele carriers already have. The results support and extend our previously finding of interaction between fibre and IL10 in a subcohort of the present study cohort [17]. Similarly, we observed interactions between dietary fibre, dietary cereals and fruit on one hand and genetic variation in *PTGS2* on the other. The interactions are quite consistent and suggest that subjects with genetically low PTGS2 activity benefit the most from high intake of fibres, fruit, and cereals. Furthermore, tertile analyses showed that those with the genetically determined lowest COX-2 activity, namely homozygous carriers of the variant allele of PTGS2 A-1195G, were at high risk of CRC in the group with the lowest intake of fibres (IRR=3.08 (95%CI:1.51-6.28), and fruits (IRR=2.11, 95%CI: 1.03-4.33), whereas those with the genetically determined high COX-2 activity, carriers of the variant allele of PTGS2 G-765C, were at low CRC risk even in the tertile with the lowest fibre intake (IRR=0.69, 95% CI: 0.50-0.96). Thus, COX-2 seems intimately implicated in the biological mechanism underlying the protective effect of fibres in relation to CRC.

We found interaction between NSAID use and *IL1B* C-3737T in relation to development of CRC suggesting that NSAID intake reduce the risk of CRC among those with high risk of CRC due to genetically determined high IL1B level. We found no statistically significant interaction between NSAID and COX-2 in relation to CRC. A non-statistically significant tendency towards protection by NSAID use among those with genetically low COX-2 activity was found. Long-term intake of aspirin (a COX-1 inhibitor) has been found to confer protection against CRC including the presently used Diet, Cancer and Health cohort [47]. It was not possible to assess the effects of specific COX-2 inhibitors due to late introduction to the Danish market and low frequency of use in the follow-up period [47,48]. 

The biological interpretation of our results is supported by other findings. IL-1B, IL-10, and COX-2 are part of the same inflammatory pathways. IL-1 has been found to induce the synthesis of COX-2 through activation of the pro-inflammatory p65 unit of nuclear factor κB (NF-κB) [49]. Furthermore, IL-10 has been found to block IL-1-induced NF-κB activation in intestinal cells (by inhibiting IκB phosphorylation) and to reduce COX-2 induction in intestinal cells [49], The latter is in accordance with the finding that *cox-2* expression is high in il-10 deficient mice [7]. Therefore, diet such as fibre may modify IL10 which act as an inflammatory “gate-keeper” and thereby affect inflammation.

Furthermore, our results suggest that those with genetically determined low COX-2 activity are at high risk of CRC by smoking and meat intake and, furthermore, protected by fibre intake. Thus *PTGS2* polymorphisms may have differential impact on CRC risk dependent on environmental factors. However, once carcinogenesis has been initiated, a high COX-2 enzyme activity seems to be a risk factor for further progression [7,50,51]. 

Taken together, our interaction analyses suggest that diet modify intestinal carcinogenesis through impact on inflammatory response and furthermore suggest that the effect may differ among various populations depending on gene-environment interactions. Our findings should be explored in other well-characterized prospective cohorts.

This study used a nested prospective case-cohort design and has the major advantage that data and samples were collected before diagnosis thus minimizing the risk of differential misclassification between cases and comparison group. The risk estimates were adjusted for known confounding factors affecting risk of CRC in this cohort including dietary factors, body mass index (BMI), alcohol, smoking status and NSAID use. A main strength of the study is the large sample size. The genes were carefully selected based on their role in the inflammatory pathway and the polymorphisms were mainly selected based on their functional effects in order to allow interpretation of the involved biological pathways in colorectal carcinogenesis. Only the interactions between fibre and *IL10*, fibre and *PTGS2*, vegetables and *IL10*, and fruit and *PTGS2* withstood correction for multiple analyses. However, as our hypothesis was biologically based we did not correct for multiple analyses [52]. In the light of the number of statistical tests performed, we would expect that some of the findings may be due to chance, but the number of statistically significant findings exceed the number expected by chance. 

## Conclusions

We found evidence that genetically determined variation in IL-1β and COX-2 levels is associated with risk of CRC. Moreover, gene-environment interactions suggest that COX-2 and IL10 are implicated in both meat-related carcinogenesis and in the protective effects of fibre in relation to CRC. This study demonstrates that gene-environment interactions provide an efficient tool for identifying factors involved in colorectal carcinogenesis. Our findings should be replicated in other well-characterized prospective cohorts.

## Supporting Information

Table S1 **Combinations of genotypes/haplotypes and risk of colorectal cancer.**
(DOCX)

Table S2 **Incidence rate ratio (IRR) for colorectal cancer for tertiles of intake of diet for the studied polymorphisms.**
(DOCX)

Table S3 **Interactions between NSAID use (no, yes) and studied polymorphisms in relation to risk of colorectal cancer.**
(DOCX)

Table S4 **Interaction between smoking status (never, past, current) and the studied polymorphisms in relation to risk of colorectal cancer.**
(DOCX)

## References

[1] Cancer World Research Fund/American Institute for CancerResearch. Available: http://www.dietandcancerreport.org/. Accessed 2013 June 16.

[2] Huxley RR, nsary-Moghaddam A, Clifton P, Czernichow S, Parr CL et al (2009) The impact of dietary and lifestyle risk factors on risk of colorectal cancer: a quantitative overview of the epidemiological evidence. Int J Cancer 125: 171-180. doi:10.1002/ijc.24343. PubMed: 19350627. 19350627

[3] Andersen V, Ostergaard M, Christensen J, Overvad K, Tjønneland A et al. (2009) Polymorphisms in the xenobiotic transporter Multidrug Resistance 1 (MDR1) gene and interaction with meat intake in relation to risk of colorectal cancer in a Danish prospective case-cohort study. BMC Cancer 9: 407. doi:10.1186/1471-2407-9-407. PubMed: 19930591. 19930591 PMC2797527

[4] Andersen V, Christensen J, Overvad K, Tjønneland A, Vogel U (2011) Heme oxygenase-1 polymorphism is not associated with risk of colorectal cancer: a Danish prospective study. Eur J Gastroenterol Hepatol 23: 282-285. doi:10.1097/MEG.0b013e3283417f76. PubMed: 21191307. 21191307

[5] Andersen V, Christensen J, Overvad K, Tjønneland A, Vogel U (2010) Polymorphisms in NFkB, PXR, LXR and risk of colorectal cancer in a prospective study of Danes. BMC Cancer 10: 484. doi:10.1186/1471-2407-10-484. PubMed: 20836841. 20836841 PMC2949803

[6] Andersen V, Holst R, Vogel U (2013) Systematic review: diet-gene interactions and the risk of colorectal cancer. Aliment Pharmacol Ther 37: 383-391. doi:10.1111/apt.12180. PubMed: 23216531. 23216531 PMC3565452

[7] Wang D, DuBois RN (2010) The role of COX-2 in intestinal inflammation and colorectal cancer. Oncogene 29: 781-788. doi:10.1038/onc.2009.421. PubMed: 19946329. 19946329 PMC3181054

[8] Erridge C (2011) Accumulation of stimulants of Toll-like receptor (TLR)-2 and TLR4 in meat products stored at 5 degrees C. J Food Sci 76: H72-H79. doi:10.1111/j.1750-3841.2010.02018.x. PubMed: 21535770. 21535770

[9] Tjonneland A, Overvad K, Bergmann MM, Nagel G, Linseisen J et al. (2009) Linoleic acid, a dietary n-6 polyunsaturated fatty acid, and the aetiology of ulcerative colitis: a nested case-control study within a European prospective cohort study. Gut 58: 1606-1611. doi:10.1136/gut.2008.169078. PubMed: 19628674. 19628674

[10] Joensen AM, Schmidt EB, Dethlefsen C, Johnsen SP, Tjønneland A et al. (2010) Dietary intake of total marine n-3 polyunsaturated fatty acids, eicosapentaenoic acid, docosahexaenoic acid and docosapentaenoic acid and the risk of acute coronary syndrome - a cohort study. Br J Nutr 103: 602-607. doi:10.1017/S0007114509992170. PubMed: 19825219. 19825219

[11] Vinolo MA, Rodrigues HG, Nachbar RT, Curi R (2011) Regulation of inflammation by short chain fatty acids. Nutrients 3: 858-876. doi:10.3390/nu3100858. PubMed: 22254083. 22254083 PMC3257741

[12] Paul G, Khare V, Gasche C (2012) Inflamed gut mucosa: downstream of interleukin-10. Eur J Clin Invest 42: 95-109. doi:10.1111/j.1365-2362.2011.02552.x. PubMed: 21631466. 21631466

[13] Berg DJ, Davidson N, Kühn R, Müller W, Menon S et al. (1996) Enterocolitis and colon cancer in interleukin-10-deficient mice are associated with aberrant cytokine production and CD4(+) TH1-like responses. J Clin Invest 98: 1010-1020. doi:10.1172/JCI118861. PubMed: 8770874. 8770874 PMC507517

[14] Vogel U, Christensen J, Wallin H, Friis S, Nexø BA et al. (2008) Polymorphisms in genes involved in the inflammatory response and interaction with NSAID use or smoking in relation to lung cancer risk in a prospective study. Mutat Res 639: 89-100. doi:10.1016/j.mrfmmm.2007.11.004. PubMed: 18164040. 18164040

[15] Vangsted AJ, Nielsen KR, Klausen TW, Haukaas E, Tjønneland A et al. (2012) A functional polymorphism in the promoter region of the IL1B gene is associated with risk of multiple myeloma. Br J Haematol 158: 515-518. doi:10.1111/j.1365-2141.2012.09141.x. PubMed: 22540426. 22540426

[16] Garcia Rodriguez LA, Cea-Soriano L, Tacconelli S, Patrignani P (2013) Coxibs: pharmacology, toxicity and efficacy in cancer clinical trials. Recent Results Cancer Res 191: 67-93. doi:10.1007/978-3-642-30331-9_4. PubMed: 22893200.: 67-93 22893200

[17] Andersen V, Egeberg R, Tjønneland A, Vogel U (2012) Interaction between interleukin-10 (IL-10) polymorphisms and dietary fibre in relation to risk of colorectal cancer in a Danish case-cohort study. BMC Cancer 12: 183. doi:10.1186/1471-2407-12-183. PubMed: 22594912.: 183-12 22594912 PMC3472168

[18] Vogel U, Christensen J, Dybdahl M, Friis S, Hansen RD et al. (2007) Prospective study of interaction between alcohol, NSAID use and polymorphisms in genes involved in the inflammatory response in relation to risk of colorectal cancer. Mutat Res 624: 88-100. doi:10.1016/j.mrfmmm.2007.04.006. PubMed: 17544013. 17544013

[19] Tjønneland A, Olsen A, Boll K, Stripp C, Christensen J et al. (2007) Study design, exposure variables, and socioeconomic determinants of participation in Diet, Cancer and Health: a population-based prospective cohort study of 57,053 men and women in Denmark. Scand J Public Health 35: 432-441. doi:10.1080/14034940601047986. PubMed: 17786808. 17786808

[20] Slimani N, Deharveng G, Unwin I, Southgate DA, Vignat J et al. (2007) The EPIC nutrient database project (ENDB): a first attempt to standardize nutrient databases across the 10 European countries participating in the EPIC study. Eur J Clin Nutr 61: 1037-1056. doi:10.1038/sj.ejcn.1602679. PubMed: 17375121. 17375121

[21] Prosky L, Asp NG, Furda I, DeVries JW, Schweizer TF et al. (1985) Determination of total dietary fiber in foods and food products: collaborative study. J Assoc Off Anal Chem 68: 677-679. PubMed: 2993226. 2993226

[22] Slimani N, Kaaks R, Ferrari P, Casagrande C, Clavel-Chapelon F et al. (2002) European Prospective Investigation into Cancer and Nutrition (EPIC) calibration study: rationale, design and population characteristics. Public Health Nutr 5: 1125-1145. doi:10.1079/PHN2002395. PubMed: 12639223. 12639223

[23] Tjønneland A, Overvad K, Haraldsdóttir J, Bang S, Ewertz M et al. (1991) Validation of a semiquantitative food frequency questionnaire developed in Denmark. Int J Epidemiol 20: 906-912. doi:10.1093/ije/20.4.906. PubMed: 1800429. 1800429

[24] Tjonneland A, Haraldsdóttir J, Overvad K, Stripp C, Ewertz M et al. (1992) Influence of individually estimated portion size data on the validity of a semiquantitative food frequency questionnaire. Int J Epidemiol 21: 770-777. doi:10.1093/ije/21.4.770. PubMed: 1521982. 1521982

[25] Miller SA, Dykes DD, Polesky HF (1988) A simple salting out procedure for extracting DNA from human nucleated cells. Nucleic Acids Res 16: 1215. doi:10.1093/nar/16.3.1215. PubMed: 3344216. 3344216 PMC334765

[26] Stegger JG, Schmidt EB, Tjønneland A, Kopp TI, Sørensen TI et al. (2012) Single nucleotide polymorphisms in IL1B and the risk of acute coronary syndrome: a Danish case-cohort study. PLOS ONE 7: e36829. doi:10.1371/journal.pone.0036829. PubMed: 22768033. 22768033 PMC3387186

[27] Barlow WE, Ichikawa L, Rosner D, Izumi S (1999) Analysis of case-cohort designs. J Clin Epidemiol 52: 1165-1172. doi:10.1016/S0895-4356(99)00102-X. PubMed: 10580779. 10580779

[28] Barlow WE (1994) Robust variance estimation for the case-cohort design. Biometrics 50: 1064-1072. doi:10.2307/2533444. PubMed: 7786988. 7786988

[29] Andersen V, Christensen J, Overvad K, Tjonneland A, Vogel U (2010) Heme oxygenase-1 (HO-1) polymorphism is not associated with risk of colorectal cancer; a Danish prospective study. Eur J Gastroenterol Hepatol. 10.1097/MEG.0b013e3283417f76 21191307

[30] Andersen V, Agerstjerne L, Jensen D, Østergaard M, Saebø M et al. (2009) The multidrug resistance 1 (MDR1) gene polymorphism G-rs3789243-A is not associated with disease susceptibility in Norwegian patients with colorectal adenoma and colorectal cancer; a case control study. BMC Med Genet 10: 18. doi:10.1186/1471-2156-10-18. PubMed: 19250544. 19250544 PMC2662819

[31] Hansen RD, Krath BN, Frederiksen K, Tjønneland A, Overvad K et al. (2009) GPX1 Pro(198)Leu polymorphism, erythrocyte GPX activity, interaction with alcohol consumption and smoking, and risk of colorectal cancer. Mutat Res 664: 13-19. doi:10.1016/j.mrfmmm.2009.01.009. PubMed: 19428376. 19428376

[32] Hansen RD, Sorensen M, Tjonneland A, Overvad K, Wallin H et al. (2008) A haplotype of polymorphisms in ASE-1, RAI and ERCC1 and the effects of tobacco smoking and alcohol consumption on risk of colorectal cancer: a Danish prospective case-cohort study. BMC Cancer %20;8:54.: 54. 10.1186/1471-2407-8-54 PMC2263058 18289367

[33] Andersen V, Egeberg R, Tjønneland A, Vogel U (2012) ABCC2 transporter gene polymorphisms, diet and risk of colorectal cancer: a Danish prospective cohort study. Scand J Gastroenterol 47: 572-574. doi:10.3109/00365521.2012.668933. PubMed: 22428913. 22428913

[34] Hansen RD, Sørensen M, Tjønneland A, Overvad K, Wallin H et al. (2007) XPA A23G, XPC Lys939Gln, XPD Lys751Gln and XPD Asp312Asn polymorphisms, interactions with smoking, alcohol and dietary factors, and risk of colorectal cancer. Mutat Res 619: 68-80. doi:10.1016/j.mrfmmm.2007.02.002. PubMed: 17363013. 17363013

[35] Vogel U, Christensen J, Wallin H, Friis S, Nexø BA et al. (2007) Polymorphisms in COX-2, NSAID use and risk of basal cell carcinoma in a prospective study of Danes. Mutat Res 617: 138-146. doi:10.1016/j.mrfmmm.2007.01.005. PubMed: 17307204. 17307204

[36] Vangsted AJ, Klausen TW, Abildgaard N, Andersen NF, Gimsing P et al. (2011) Single nucleotide polymorphisms in the promoter region of the IL1B gene influence outcome in multiple myeloma patients treated with high-dose chemotherapy independently of relapse treatment with thalidomide and bortezomib. Ann Hematol, 90: 1173–81. PubMed: 21347685. 21347685 10.1007/s00277-011-1194-3

[37] Ravn-Haren G, Olsen A, Tjønneland A, Dragsted LO, Nexø BA et al. (2006) Associations between GPX1 Pro198Leu polymorphism, erythrocyte GPX activity, alcohol consumption and breast cancer risk in a prospective cohort study. Carcinogenesis 27: 820-825. PubMed: 16287877. 16287877 10.1093/carcin/bgi267

[38] Language A and Environment for Statistical Computing (2004). Available: http://www.R-project.org. Accessed 2013 June 16. .

[39] Chen H, Wilkins LM, Aziz N, Cannings C, Wyllie DH et al. (2006) Single nucleotide polymorphisms in the human interleukin-1B gene affect transcription according to haplotype context. Hum Mol Genet 15: 519-529. doi:10.1093/hmg/ddi469. PubMed: 16399797. 16399797

[40] Agudo A, Slimani N, Ocké MC, Naska A, Miller AB et al. (2002) Consumption of vegetables, fruit and other plant foods in the European Prospective Investigation into Cancer and Nutrition (EPIC) cohorts from 10 European countries. Public Health Nutr 5: 1179-1196. doi:10.1079/PHN2002398. PubMed: 12639226. 12639226

[41] Zhang X, Miao X, Tan W, Ning B, Liu Z et al. (2005) Identification of functional genetic variants in cyclooxygenase-2 and their association with risk of esophageal cancer. Gastroenterology 129: 565-576. doi:10.1016/j.gastro.2005.05.003. PubMed: 16083713. 16083713

[42] Moore AE, Young LE, Dixon DA (2012) A common single-nucleotide polymorphism in cyclooxygenase-2 disrupts microRNA-mediated regulation. Oncogene 31: 1592-1598. doi:10.1038/onc.2011.349. PubMed: 21822307. 21822307 PMC3454533

[43] Sanak M, Szczeklik W, Szczeklik A (2005) Association of COX-2 gene haplotypes with prostaglandins production in bronchial asthma. J Allergy Clin Immunol 116: 221-223. doi:10.1016/j.jaci.2005.03.010. PubMed: 15990799. 15990799

[44] Andersen V, Nimmo E, Krarup HB, Drummond H, Christensen J et al. (2011) Cyclooxygenase-2 (COX-2) polymorphisms and risk of inflammatory bowel disease in a Scottish and Danish case-control study. Inflamm Bowel Dis 17: 937-946. doi:10.1002/ibd.21440. PubMed: 20803508. 20803508

[45] Koh WP, Yuan JM, van den BD, Lee HP, Yu MC (2004) Interaction between cyclooxygenase-2 gene polymorphism and dietary n-6 polyunsaturated fatty acids on colon cancer risk: the Singapore Chinese Health Study. Br J Cancer 90: 1760-1764. PubMed: 15150618. 15150618 10.1038/sj.bjc.6601797 PMC2409730

[46] Franke A, Balschun T, Karlsen TH, Sventoraityte J, Nikolaus S et al. (2008) Sequence variants in IL10, ARPC2 and multiple other loci contribute to ulcerative colitis susceptibility. Nat Genet 40: 1319-1323. doi:10.1038/ng.221. PubMed: 18836448. 18836448

[47] Friis S, Poulsen AH, Sørensen HT, Tjønneland A, Overvad K et al. (2009) Aspirin and other non-steroidal anti-inflammatory drugs and risk of colorectal cancer: a Danish cohort study. Cancer Causes Control 20: 731-740. doi:10.1007/s10552-008-9286-7. PubMed: 19122977. 19122977

[48] Flossmann E, Rothwell PM (2007) Effect of aspirin on long-term risk of colorectal cancer: consistent evidence from randomised and observational studies. Lancet 369: 1603-1613. doi:10.1016/S0140-6736(07)60747-8. PubMed: 17499602. 17499602

[49] Al-Ashy R, Chakroun I, El-Sabban ME, Homaidan FR (2006) The role of NF-kappaB in mediating the anti-inflammatory effects of IL-10 in intestinal epithelial cells. Cytokine 36: 1-8. doi:10.1016/j.cyto.2006.10.003. PubMed: 17161612. 17161612

[50] Al-Salihi MA, Terrece PA, Doan T, Reichert EC, Rosenberg DW et al. (2009) Transgenic expression of cyclooxygenase-2 in mouse intestine epithelium is insufficient to initiate tumorigenesis but promotes tumor progression. Cancer Lett 273: 225-232. doi:10.1016/j.canlet.2008.08.012. PubMed: 18790560. 18790560 PMC2646674

[51] Hedlund M, Padler-Karavani V, Varki NM, Varki A (2008) Evidence for a human-specific mechanism for diet and antibody-mediated inflammation in carcinoma progression. Proc Natl Acad Sci U S A 105: 18936-18941. doi:10.1073/pnas.0803943105. PubMed: 19017806. 19017806 PMC2596253

[52] Perneger TV (1998) What's wrong with Bonferroni adjustments. BMJ 316: 1236-1238. doi:10.1136/bmj.316.7139.1236. PubMed: 9553006. 9553006 PMC1112991

