# Supplementary material for: Interactions between Diet, Lifestyle and IL10, IL1B, and PTGS2/COX-2 Gene Polymorphisms in Relation to Risk of Colorectal Cancer in a Prospective Danish Case-Cohort Study
Source: PLoS One. 2013 Oct 23;8(10):e78366. doi: 10.1371/journal.pone.0078366 (PMC3806836; doi:10.1371/journal.pone.0078366)
Supplement: Table S1 — Combinations of genotypes/haplotypes and risk of colorectal cancer. (DOCX) [file pone.0078366.s001.docx]

Table S1. Combinations of genotypes/haplotypes and risk of colorectal cancer

|  |  | N_cases_ | N_subcohort_ | IRR^a^ | (95%CI) | IRR^b^ | (95%CI) | P-value^c^ |
| --- | --- | --- | --- | --- | --- | --- | --- | --- |
| *IL1B* | TGT/TGT | 168 | 353 | 1 |  | 1 |  | 1 |
|  | TGT/CCT | 227 | 419 | 1.13 | (0.88-1.45) | 1.11 | (0.86-1.43) | 0.44 |
|  | TGT/CGT | 159 | 326 | 1.05 | (0.80-1.37) | 1.01 | (0.77-1.33) | 0.94 |
|  | TGT/CGC | 35 | 94 | 0.79 | (0.51-1.22) | 0.76 | (0.49-1.19) | 0.23 |
|  | CCC/CCC | 81 | 139 | 1.28 | (0.91-1.79) | 1.28 | (0.91-1.82) | 0.16 |
|  | CCC/GCT | 137 | 209 | 1.39 | (1.04-1.86) | 1.41 | (1.05-1.89) | 0.02 |
|  | CCC/CGC | 33 | 59 | 1.34 | (0.84-2.15) | 1.25 | (0.77-2.03) | 0.37 |
|  | CGT/CGT | 48 | 97 | 1.06 | (0.71-1.58) | 1.06 | (0.71-1.59) | 0.78 |
|  | CGT/CGC | 27 | 52 | 1.11 | (0.67-1.85) | 1.10 | (0.65-1.85) | 0.72 |
|  | CGC/CGC | 3 | 6 | 1.22 | (0.29-5.07) | 1.00 | (0.24-4.24) | 1.00 |
|  |  |  |  |  |  |  |  |  |
| *IL10* | CC CC^d^ | 352 | 646 | 1 |  | 1 |  | 1 |
|  | CC CT | 200 | 391 | 0.96 | (0.77-1.20) | 0.97 | (0.77-1.21) | 0.77 |
|  | CC TT | 34 | 52 | 1.07 | (0.67-1.70) | 1.03 | (0.64-1.65) | 0.90 |
|  | AC CC | 234 | 460 | 0.94 | (0.76-1.15) | 0.93 | (0.75-1.15) | 0.51 |
|  | AC CT | 62 | 128 | 0.89 | (0.63-1.25) | 0.91 | (0.64-1.28) | 0.57 |
|  | AA CC | 56 | 96 | 1.02 | (0.71-1.47) | 1.00 | (0.69-1.45) | 0.98 |
|  |  |  |  |  |  |  |  |  |
| *PTGS2* | AGT/AGT | 185 | 343 | 1 |  | 1 |  | 1 |
|  | AGT/GGT | 175 | 299 | 1.05 | (0.81-1.37) | 1.08 | (0.83-1.42) | 0.56 |
|  | AGT/AGC | 160 | 314 | 0.93 | (0.71-1.22) | 0.94 | (0.72-1.24) | 0.67 |
|  | AGT/ACC | 109 | 225 | 0.91 | (0.68-1.22) | 0.89 | (0.66-1.21) | 0.46 |
|  | GGT/GGT | 46 | 57 | 1.47 | (0.94-2.28) | 1.48 | (0.95-2.32) | 0.09 |
|  | GGT/AGC | 71 | 160 | 0.82 | (0.59-1.16) | 0.81 | (0.57-1.15) | 0.24 |
|  | GGT/ACC | 47 | 95 | 0.96 | (0.64-1.44) | 0.90 | (0.60-1.37) | 0.63 |
|  | AGC/AGC | 34 | 71 | 0.82 | (0.51-1.29) | 0.83 | (0.52-1.33) | 0.45 |
|  | AGC/ACC | 42 | 95 | 0.77 | (0.51-1.16) | 0.73 | (0.48-1.10) | 0.13 |
|  | ACC/ACC | 19 | 35 | 0.97 | (0.54-1.76) | 1.00 | (0.55-1.83) | 0.99 |

Genotype and haplotype sequence: *IL10*: C-592A, C-rs3024505-T, *IL1B* C-3737T, G-1464C, T-31C, *PTGS2* A-1195G, G-765C, T8473C

^a^ Adjusted for sex and age

^b^ In addition, adjusted for smoking status, alcohol, HRT status (women only), BMI, intake of red and processed meat, and dietary fibre

^c^ P p-value for interaction for the adjusted risk estimates

^d^ Genotype combinations for *IL10*: C-592A and C-rs3024505-T
