# Supplementary material for: Interactions between Diet, Lifestyle and IL10, IL1B, and PTGS2/COX-2 Gene Polymorphisms in Relation to Risk of Colorectal Cancer in a Prospective Danish Case-Cohort Study
Source: PLoS One. 2013 Oct 23;8(10):e78366. doi: 10.1371/journal.pone.0078366 (PMC3806836; doi:10.1371/journal.pone.0078366)
Supplement: Table S2 — Incidence rate ratio (IRR) for colorectal cancer for tertiles of intake of diet for the studied polymorphisms. (DOCX) [file pone.0078366.s002.docx]

Table S2. Incidence rate ratio (IRR) for colorectal cancer for tertiles of intake of diet for the studied polymorphisms.

|  | 1.tertile | | 2.tertile | | 3.tertile | | 1.tertile | 2.tertile | 3.tertile | Pint | 1.tertile | | 2.tertile | | 3.tertile | | 1.tertile | 2.tertile | 3.tertile | Pint |
| --- | --- | --- | --- | --- | --- | --- | --- | --- | --- | --- | --- | --- | --- | --- | --- | --- | --- | --- | --- | --- |
|  | Nc | Ns | Nc | Ns | Nc | Ns | IRR (95%CI) | IRR (95%CI) | IRR (95%CI) |  | Nc | Ns | Nc | Ns | Nc | Ns | IRR (95%CI) | IRR (95%CI) | IRR (95%CI) |  |
| ***IL10* C-592A** | | | | | | | red and processed meat | | |  |  |  |  |  |  |  | Fish | | |  |
| CC | 170 | 366 | 208 | 364 | 218 | 361 | 1.00 | 1.31 (1.00-1.70) | 1.34 (1.02-1.77) |  | 193 | 359 | 210 | 346 | 193 | 386 | 1.00 | 1.10 (0.85-1.42) | 0.89 (0.69-1.16) |  |
| AC-AA | 114 | 248 | 118 | 209 | 121 | 223 | 0.99 (0.74-1.34) | 1.25 (0.92-1.69) | 1.16 (0.84-1.60) | 0.703 | 110 | 239 | 128 | 210 | 115 | 231 | 0.89 (0.66-1.20) | 1.12 (0.83-1.50) | 0.79 (0.59-1.07) | 0.658 |
|  |  |  |  |  |  |  | dietary fibre | | |  |  |  |  |  |  |  | fruit | | |  |
| CC | 202 | 356 | 212 | 344 | 182 | 391 | 1.00 | 1.17 (0.91-1.51) | 0.92 (0.71-1.20) |  | 207 | 355 | 198 | 336 | 191 | 400 | 1.00 | 1.04 (0.81-1.35) | 0.84 (0.65-1.10) |  |
| AC-AA | 132 | 214 | 113 | 233 | 108 | 233 | 1.18 (0.88-1.58) | 0.89 (0.66-1.20) | 0.81 (0.60-1.10) | 0.025 | 122 | 217 | 129 | 232 | 102 | 231 | 1.00 (0.75-1.34) | 0.98 (0.73-1.30) | 0.70 (0.52-0.96) | 0.557 |
|  |  |  |  |  |  |  | cereal | | |  |  |  |  |  |  |  | vegetables | | |  |
| CC | 215 | 357 | 190 | 357 | 191 | 377 | 1.00 | 0.91 (0.70-1.17) | 0.88 (0.67-1.14) |  | 212 | 339 | 198 | 367 | 186 | 385 | 1.00 | 1.01 (0.78-1.31) | 0.90 (0.69-1.18) |  |
| AC-AA | 121 | 204 | 115 | 242 | 117 | 234 | 1.00 (0.75-1.35) | 0.78 (0.59-1.05) | 0.82 (0.60-1.11) | 0.663 | 129 | 217 | 108 | 230 | 116 | 233 | 1.03 (0.77-1.38) | 0.81 (0.60-1.08) | 0.88 (0.65-1.19) | 0.258 |
| **IL10 rs3024505** | | | | | | | red and processed meat | | |  |  |  |  |  |  |  | fish | | |  |
| CC | 193 | 414 | 232 | 388 | 223 | 413 | 1.00 | 1.36 (1.06-1.75) | 1.17 (0.90-1.54) |  | 210 | 408 | 230 | 374 | 208 | 433 | 1.00 | 1.13 (0.89-1.45) | 0.84 (0.65-1.07) |  |
| CT-TT | 85 | 206 | 95 | 191 | 117 | 176 | 0.89 (0.65-1.23) | 1.12 (0.81-1.53) | 1.50 (1.09-2.06) | 0.022 | 86 | 198 | 109 | 185 | 102 | 190 | 0.85 (0.62-1.16) | 1.13 (0.84-1.53) | 0.94 (0.69-1.27) | 0.286 |
|  |  |  |  |  |  |  | dietary fibre | | |  |  |  |  |  |  |  | fruit | | |  |
| CC | 246 | 393 | 211 | 398 | 191 | 424 | 1.00 | 0.84 (0.66-1.07) | 0.73 (0.57-0.94) |  | 234 | 400 | 218 | 378 | 196 | 437 | 1.00 | 0.98 (0.76-1.25) | 0.74 (0.57-0.95) |  |
| CT-TT | 88 | 183 | 111 | 181 | 98 | 209 | 0.73 (0.54-1.01) | 1.05 (0.78-1.41) | 0.77 (0.57-1.04) | 0.007 | 96 | 176 | 106 | 195 | 95 | 202 | 0.91 (0.67-1.23) | 0.96 (0.71-1.30) | 0.79 (0.58-1.08) | 0.629 |
|  |  |  |  |  |  |  | cereal | | |  |  |  |  |  |  |  | vegetables | | |  |
| CC | 234 | 386 | 203 | 409 | 211 | 420 | 1.00 | 0.82 (0.64-1.05) | 0.85 (0.65-1.10) |  | 248 | 379 | 218 | 412 | 182 | 424 | 1.00 | 0.88 (0.69-1.11) | 0.72 (0.56-0.93) |  |
| CT-TT | 101 | 182 | 100 | 193 | 96 | 198 | 0.96 (0.70-1.30) | 0.88 (0.65-1.19) | 0.81 (0.59-1.10) | 0.755 | 91 | 184 | 87 | 190 | 119 | 199 | 0.75 (0.55-1.02) | 0.78 (0.57-1.07) | 1.03 (0.77-1.38) | 0.001 |
| ***PTGS2* A-1195G** | | | | | | | red and processed meat | | |  |  |  |  |  |  |  | fish | | |  |
| AA-AG | 268 | 594 | 311 | 552 | 321 | 562 | 1.00 | 1.31 (1.05-1.62) | 1.27 (1.01-1.60) |  | 289 | 583 | 322 | 531 | 289 | 594 | 1.00 | 1.18 (0.96-1.45) | 0.89 (0.72-1.10) |  |
| GG | 13 | 20 | 18 | 23 | 16 | 19 | 1.38 (0.65-12.94) | 1.83 (0.95-3.54) | 1.82 (0.90-3.70) | 0.995 | 14 | 18 | 14 | 21 | 19 | 23 | 1.88 (0.88-4.04) | 1.28 (0.61-2.66) | 1.33 (0.71-2.51) | 0.348 |
|  |  |  |  |  |  |  | dietary fibre | | |  |  |  |  |  |  |  | fruit | | |  |
| AA-AG | 317 | 555 | 309 | 552 | 274 | 601 | 1.00 | 1.02 (0.83-1.26) | 0.84 (0.68-1.04) |  | 313 | 554 | 313 | 541 | 274 | 613 | 1.00 | 1.04 (0.85-1.29) | 0.78 (0.63-0.97) |  |
| GG | 20 | 16 | 13 | 23 | 14 | 23 | 3.08 (1.51-6.28) | 0.88 (0.42-1.81) | 1.01 (0.51-2.02) | 0.002 | 18 | 16 | 15 | 27 | 14 | 19 | 2.11 (1.03-4.33) | 0.98 (0.51-1.90) | 1.17 (0.55-2.49) | 0.080 |
|  |  |  |  |  |  |  | Cereal | | |  |  |  |  |  |  |  | vegetables | | |  |
| AA-AG | 319 | 544 | 284 | 575 | 297 | 589 | 1.00 | 0.84 (-0.69-1.04) | 0.87 (0.70-1.09) |  | 326 | 537 | 290 | 579 | 284 | 592 | 1.00 | 0.92 (0.75-1.13) | 0.90 (0.72-1.12) |  |
| GG | 17 | 16 | 19 | 22 | 11 | 24 | 1.82 (0.87-3.84) | 1.51 (0.77-2.99) | 0.73 (0.36-1.50) | 0.074 | 17 | 19 | 13 | 18 | 17 | 25 | 1.84 (0.91-3.70) | 1.25 (0.58-2.69) | 1.08 (0.56-2.06) | 0.480 |
| ***PTGS2* G-765C** | | | | | | | red and processed meat | | |  |  |  |  |  |  |  | fish | | |  |
| GG | 219 | 440 | 249 | 411 | 233 | 422 | 1.00 | 1.28 (1.01-1.62) | 1.10 (0.85-1.42) |  | 227 | 421 | 246 | 411 | 228 | 441 | 1.00 | 1.06 (0.84-1.34) | 0.85 (0.67-1.07) |  |
| CG-CC | 60 | 165 | 71 | 158 | 104 | 161 | 0.74 (0.52-1.05) | 0.91 (0.64-1.28) | 1.33 (0.96-1.84) | 0.0048 | 69 | 173 | 87 | 141 | 79 | 170 | 0.72 (0.51-1.01) | 1.09 (0.79-1.51) | 0.78 (0.57-1.08) | 0.143 |
|  |  |  |  |  |  |  | dietary fibre | | |  |  |  |  |  |  |  | fruit | | |  |
| GG | 255 | 400 | 238 | 406 | 208 | 467 | 1.00 | 0.94 (0.74-1.20) | 0.71 (0.55-0.90) |  | 241 | 406 | 247 | 397 | 213 | 470 | 1.00 | 1.07 (0.85-1.37) | 0.73 (0.57-0.93) |  |
| CG-CC | 75 | 169 | 78 | 161 | 82 | 154 | 0.69 (0.50-0.96) | 0.76 (0.55-1.05) | 0.89 (0.64-1.24) | 0.004 | 87 | 164 | 70 | 165 | 78 | 155 | 0.88 (0.64-1.20) | 0.70 (0.50-0.98) | 0.87 (0.62-1.21) | 0.006 |
|  |  |  |  |  |  |  | cereal | | |  |  |  |  |  |  |  | vegetables | | |  |
| GG | 249 | 403 | 234 | 415 | 218 | 455 | 1.00 | 0.90 (0.71-1.14) | 0.78 (0.61-1.00) |  | 250 | 405 | 235 | 410 | 216 | 458 | 1.00 | 1.02 (0.80-1.29) | 0.85 (0.66-1.08) |  |
| CG-CC | 80 | 153 | 70 | 175 | 85 | 156 | 0.85 (0.61-1.18) | 0.65 (0.47-0.91) | 0.87 (0.62-1.21) | 0.0670 | 84 | 154 | 69 | 177 | 82 | 153 | 0.86 (0.62-1.18) | 0.70 (0.50-0.97) | 0.99 (0.71-1.38) | 0.020 |
| ***PTGS2* T8473C** | | | | | | | red and processed meat | | |  |  |  |  |  |  |  | fish | | |  |
| TT | 118 | 259 | 162 | 238 | 150 | 235 | 1.00 | 1.61 (1.18-2.19) | 1.44 (1.04-1.99) |  | 131 | 255 | 156 | 233 | 143 | 244 | 1.00 | 1.24 (0.92-1.68) | 1.01 (0.74-1.37) |  |
| CT-CC | 160 | 350 | 157 | 334 | 184 | 344 | 1.02 (0.76-1.38) | 1.10 (0.80-1.49) | 1.21 (0.88-1.65) | 0.05 | 163 | 343 | 179 | 315 | 159 | 370 | 0.91 (0.68-1.23) | 1.06 (0.79-1.41) | 0.74 (0.55-1.00) | 0.430 |
|  |  |  |  |  |  |  | dietary fibre | | |  |  |  |  |  |  |  | fruit | | |  |
| TT | 152 | 231 | 147 | 225 | 131 | 276 | 1.00 | 1.01(0.74-1.38) | 0.74 (0.54-1.00) |  | 144 | 221 | 155 | 241 | 131 | 270 | 1.00 | 1.05 (0.77-1.43) | 0.72 (0.52-0.99) |  |
| CT-CC | 176 | 334 | 169 | 349 | 156 | 345 | 0.80 (0.60-1.06) | 0.75 (0.56-1.00) | 0.71 (0.53-0.96) | 0.252 | 174 | 345 | 170 | 323 | 157 | 360 | 0.79 (0.59-1.05) | 0.81 (0.60-1.09) | 0.67 (0.50-0.91) | 0.420 |
|  |  |  |  |  |  |  | cereal | | |  |  |  |  |  |  |  | vegetables | | |  |
| TT | 151 | 231 | 144 | 241 | 135 | 260 | 1.00 | 0.95 (0.70-1.29) | 0.81 (0.60-1.12) |  | 150 | 230 | 146 | 232 | 134 | 270 | 1.00 | 1.06 (0.78-1.43) | 0.83 (0.61-1.14) |  |
| CT-CC | 185 | 325 | 151 | 356 | 165 | 347 | 0.90 (0.67-1.20) | 0.65 (0.48-0.87) | 0.75 (0.55-1.02) | 0.1345 | 184 | 327 | 151 | 353 | 166 | 348 | 0.85 (0.64-1.14) | 0.72 (0.54-0.96) | 0.81 (0.60-1.09) | 0.095 |
| ***IL1B* C-3737T** | | | | | | | red and processed meat | | |  |  |  |  |  |  |  | fish | | |  |
| CC | 97 | 199 | 122 | 182 | 117 | 188 | 1.00 | 1.35 (0.95-1.93) | 1.32 (0.92-1.89) |  | 104 | 206 | 124 | 163 | 108 | 200 | 1.00 | 1.52 (1.08-2.16) | 1.02 (0.72-1.45) |  |
| CT-TT | 183 | 416 | 204 | 393 | 218 | 391 | 0.84 (0.62-1.15) | 1.07 (0.78-1.46) | 1.08 (0.78-1.49) | 0.928 | 196 | 394 | 212 | 393 | 197 | 413 | 0.99 (0.73-1.34) | 1.01 (0.75-1.36) | 0.83 (0.61-1.13) | 0.056 |
|  |  |  |  |  |  |  | dietary fibre | | |  |  |  |  |  |  |  | fruit | | |  |
| GG | 121 | 186 | 124 | 195 | 91 | 188 | 1.00 | 0.95 (0.68-1.33) | 0.74 (0.52-1.06) |  | 113 | 189 | 119 | 181 | 104 | 199 | 1.00 | 1.12 (0.79-1.57) | 0.86 (0.60-1.22) |  |
| AG-AA | 213 | 382 | 198 | 379 | 194 | 439 | 0.78 (0.58-1.04) | 0.77 (0.57-1.03) | 0.65 (0.48-0.88) | 0.745 | 215 | 376 | 208 | 392 | 182 | 432 | 0.92 (0.68-1.24) | 0.86 (0.63-1.16) | 0.66 (0.49-0.89) | 0.474 |
|  |  |  |  |  |  |  | cereal | | |  |  |  |  |  |  |  | vegetables | | |  |
| GG | 110 | 183 | 117 | 201 | 109 | 185 | 1.00 | 0.93 (0.66-1.32) | 0.97 (0.67-1.40) |  | 131 | 173 | 107 | 189 | 98 | 207 | 1.00 | 0.80 (0.57-1.13) | 0.65 (0.46-0.93) |  |
| AG-AA | 223 | 376 | 189 | 398 | 193 | 426 | 0.91 (0.67-1.23) | 0.74 (0.54-1.01) | 0.71 (0.52-0.98) | 0.4512 | 209 | 380 | 195 | 405 | 201 | 415 | 0.66 (0.49-0.89) | 0.64 (0.48-0.86) | 0.67 (0.50-0.91) | 0.030 |
| ***IL1B* G-1464C** | | | | | | | red and processed meat | | |  |  |  |  |  |  |  | fish | | |  |
| GG | 136 | 318 | 146 | 304 | 172 | 315 | 1.00 | 1.24 (0.92-1.67) | 1.32 (0.97-1.78) |  | 152 | 315 | 157 | 304 | 145 | 318 | 1.00 | 1.07 (0.80-1.42) | 0.86 (0.64-1.14) |  |
| CG-CC | 147 | 298 | 178 | 270 | 167 | 267 | 1.22 (0.91-1.63) | 1.60 (1.20-2.14) | 1.51 (1.11-2.05) | 0.7450 | 148 | 286 | 181 | 250 | 163 | 299 | 1.11 (0.83-1.49) | 1.43 (1.08-1.90) | 1.04 (0.78-1.39) | 0.516 |
|  |  |  |  |  |  |  | dietary fibre | | |  |  |  |  |  |  |  | fruit | | |  |
| GG | 162 | 306 | 152 | 294 | 140 | 337 | 1.00 | 1.01 (0.76-1.35) | 0.84 (0.63-1.13) |  | 160 | 297 | 156 | 329 | 138 | 311 | 1.00 | 0.92 (0.70-1.23) | 0.82 (0.61-1.10) |  |
| CG-CC | 173 | 264 | 171 | 279 | 148 | 292 | 1.29 (0.98-1.72) | 1.22 (0.92-1.62) | 0.98 (0.73-1.31) | 0.7889 | 170 | 270 | 169 | 242 | 153 | 323 | 1.22 (0.92-1.62) | 1.31 (0.98-1.76) | 0.88 (0.66-1.18) | 0.235 |
|  |  |  |  |  |  |  | cereal | | |  |  |  |  |  |  |  | vegetables | | |  |
| GG | 165 | 288 | 139 | 314 | 150 | 335 | 1.00 | 0.83 (0.62-1.10) | 0.81 (0.60-1.08) |  | 158 | 299 | 158 | 319 | 138 | 319 | 1.00 | 1.09 (0.82-1.44) | 0.96 (0.71-1.28) |  |
| CG-CC | 172 | 275 | 165 | 285 | 155 | 275 | 1.18 (0.89-1.56) | 1.01 (0.76-1.35) | 1.02 (0.76-1.38) | 0.899 | 184 | 256 | 145 | 277 | 163 | 302 | 1.47 (1.11-1.95) | 1.11 (0.83-1.48) | 1.15 (0.86-1.54) | 0.074 |
| ***IL1B* T-31C** | | | | | | | red and processed meat | | |  |  |  |  |  |  |  | fish | | |  |
| TT | 118 | 268 | 136 | 262 | 135 | 254 | 1.00 | 1.33 (0.97-1.83) | 1.28 (0.92-1.78) |  | 135 | 259 | 134 | 255 | 120 | 270 | 1.00 | 1.01 (0.74-1.38) | 0.79 (0.58-1.08) |  |
| CT-CC | 163 | 349 | 191 | 315 | 203 | 331 | 1.13 (0.84-1.52) | 1.46 (1.08-1.96) | 1.47 (1.08-2.00) | 0.943 | 166 | 344 | 205 | 301 | 186 | 350 | 0.96 (0.72-1.28) | 1.26 (0.94-1.67) | 0.94 (0.70-1.25) | 0.248 |
|  |  |  |  |  |  |  | dietary fibre | | |  |  |  |  |  |  |  |  | | |  |
| TT | 141 | 254 | 125 | 250 | 123 | 280 | 1.00 | 0.96 (0.70-1.31) | 0.89 (0.65-1.22) |  | 135 | 249 | 134 | 269 | 120 | 266 | 1.00 | 0.98 (0.72-1.34) | 0.86 (0.62-1.18) |  |
| CT-CC | 195 | 320 | 197 | 326 | 165 | 349 | 1.18 (0.89-1.56) | 1.17 (0.88-1.55) | 0.88 (0.66-1.18) | 0.431 | 194 | 322 | 192 | 303 | 171 | 370 | 1.19 (0.89-1.58) | 1.21 (0.90-1.62) | 0.85 (0.64-1.14) | 0.379 |
|  |  |  |  |  |  |  | cereal | | |  |  |  |  |  |  |  | vegetables | | |  |
| TT | 144 | 238 | 122 | 267 | 123 | 279 | 1.00 | 0.83 (0.60-1.13) | 0.78 (0.57-1.07) |  | 138 | 259 | 134 | 259 | 117 | 266 | 1.00 | 1.17 (0.86-1.58) | 0.99 (0.72-1.36) |  |
| CT-CC | 193 | 326 | 183 | 334 | 181 | 335 | 1.07 (0.80-1.42) | 0.92 (0.69-1.24) | 0.94 (0.69-1.27) | 0.771 | 204 | 301 | 170 | 339 | 183 | 355 | 1.40 (1.05-1.86) | 1.06 (0.80-1.41) | 1.11 (0.83-1.48) | 0.028 |

^a^ Analysis adjusted for smoking status, alcohol, HRT status (women only), BMI, use of NSAID, and intake of red and processed meat,

^b^ P-value for interaction between polymorphisms and dietary factors for the adjusted estimates

Tertiles of red and processed meat (<90.7 g, 90.7 g < and < 133.5 g, > 133.5 g), fish (<29.2 g, 29.2 g < and < 48.7 g, > 48.7 g), dietary fibre (<17.5 g, 17.5 g < and < 23.0 g, > 23.0 g), fruit (<118.6 g, 118.6 g < and < 233.1 g, > 233.1 g), cereal (<154.9 g, 154.9 g < and < 218.5 g, > 218.5 g), vegetables(<118.3 g, 118.3 g < and < 199.8 g, > 199.8 g).
