# Supplementary material for: Interactions between Diet, Lifestyle and IL10, IL1B, and PTGS2/COX-2 Gene Polymorphisms in Relation to Risk of Colorectal Cancer in a Prospective Danish Case-Cohort Study
Source: PLoS One. 2013 Oct 23;8(10):e78366. doi: 10.1371/journal.pone.0078366 (PMC3806836; doi:10.1371/journal.pone.0078366)
Supplement: Table S3 — Interactions between NSAID use (no, yes) and studied polymorphisms in relation to risk of colorectal cancer. (DOCX) [file pone.0078366.s003.docx]

Table S3. Interactions between NSAID use (no, yes) and studied polymorphisms in relation to risk of colorectal cancer.

|  |  |  | NSAID No | | | | | | | | **NSAID Yes** | | | | | | | | **P-value** |
| --- | --- | --- | --- | --- | --- | --- | --- | --- | --- | --- | --- | --- | --- | --- | --- | --- | --- | --- | --- |
|  |  |  | **N_cases_** | **N_subcohort_** | **IRR^a^** | **(95%CI)** |  | **IRR^b^** | **(95%CI)** |  | N_cases_ | N_subcohort_ | **IRR^a^** | **(95%CI)** |  | **IRR^b^** | **(95%CI)** |  |  |
| ***IL10*** | C-592A | CC | 421 | 750 | 1.00 |  |  | 1.00 |  |  | 172 | 334 | 0.96 | (0.76-1.20) | | 0.97 | (0.77-1.21) | |  |
|  |  | AC-AA | 230 | 460 | 0.89 | (0.73-1.09) | | 0.91 | (0.74-1.12) | | 118 | 217 | 0.99 | (0.76-1.29) | | 0.96 | (0.73-1.25) | | 0.58 |
|  | rs3024505 | CC | 432 | 840 | 1.00 |  |  | 1.00 |  |  | 210 | 366 | 1.14 | (0.93-1.41) | | 1.11 | (0.90-1.38) | |  |
|  |  | CT-TT | 214 | 384 | 1.07 | (0.87-1.32) | | 1.08 | (0.87-1.33) | | 81 | 188 | 0.88 | (0.66--1.18) | | 0.89 | (0.66-1.19) | | 0.05 |
| ***IL1B*** | C-3737T | CC | 235 | 376 | 1.00 |  |  | 1.00 |  |  | 99 | 189 | 0.87 | (0.65-1.18) | | 0.82 | (0.61-1.12) | |  |
|  |  | CT-TT | 409 | 834 | 0.76 | (0.62-0.93) | | 0.74 | (0.60-0.91) | | 190 | 361 | 0.84 | (0.66-1.08) | | 0.82 | (0.64-1.06) | | 0.04 |
|  | G-1464C | GG | 322 | 652 | 1.00 |  |  | 1.00 |  |  | 129 | 279 | 0.99 | (0.77-1.28) | | 0.97 | (0.75-1.25) | |  |
|  |  | GC-CC | 326 | 561 | 1.19 | (0.98-1.45) | | 1.20 | (0.98-1.46) | | 161 | 270 | 1.25 | (0.98-1.59) | | 1.24 | (0.97-1.58) | | 0.65 |
|  | T-31C | TT | 281 | 545 | 1.00 |  |  | 1.00 |  |  | 105 | 233 | 0.91 | (0.69-1.20) | | 0.90 | (0.68-1.19) | |  |
|  |  | TC-CC | 369 | 672 | 1.07 | (0.88-1.30) | | 1.07 | (0.88-1.31) | | 183 | 319 | 1.16 | (0.91-1.47) | | 1.13 | (0.89-1.44) | | 0.27 |
| ***PTGS2*** | A-1195G | AA-AG | 618 | 1169 | 1.00 |  |  | 1.00 |  |  | 16 | 19 | 1.01 | (0.85-1.21) | | 0.99 | (0.83-1.19) | |  |
|  |  | GG | 31 | 43 | 1.23 | (0.76-1.99) | | 1.27 | (0.78-2.06) | | 274 | 529 | 1.47 | (0.63-3.44) | | 1.86 | (0.89-3.88) | | 0.39 |
|  | G-765C | GG | 477 | 874 | 1.00 |  |  | 1.00 |  |  | 218 | 391 | 1.09 | (0.89-1.34) | | 1.07 | (0.87-1.32) | |  |
|  |  | GC-CC | 165 | 330 | 0.96 | (0.77-1.20) | | 0.93 | (0.74-1.17) | | 68 | 152 | 0.84 | (0.61-1.15) | | 0.80 | (0.58-1.10) | | 0.18 |
|  | T8473C | TT | 292 | 514 | 1.00 |  |  | 1.00 |  |  | 134 | 215 | 1.12 | (0.86-1.46) | | 1.14 | (0.87-1.49) | |  |
|  |  | TC-CC | 346 | 695 | 0.88 | (0.72-1.07) | | 0.87 | (0.71-1.06) | | 151 | 326 | 0.87 | (0.68-1.11) | | 0.82 | (0.64-1.06) | | 0.21 |

^a^ Adjusted for sex and age

^b^ In addition, adjusted for smoking status, alcohol, HRT status (women only), BMI, intake of red and processed meat, and dietary fibre

^c^ P-value for interaction between the polymorphisms and NSAID use for the adjusted risk estimates
