# Supplementary material for: Interactions between Diet, Lifestyle and IL10, IL1B, and PTGS2/COX-2 Gene Polymorphisms in Relation to Risk of Colorectal Cancer in a Prospective Danish Case-Cohort Study
Source: PLoS One. 2013 Oct 23;8(10):e78366. doi: 10.1371/journal.pone.0078366 (PMC3806836; doi:10.1371/journal.pone.0078366)
Supplement: Table S4 — Interaction between smoking status (never, past, current) and the studied polymorphisms in relation to risk of colorectal cancer. (DOCX) [file pone.0078366.s004.docx]

Table S4. Interaction between smoking status (never, past, current) and the studied polymorphisms in relation to risk of colorectal cancer.

|  |  | **Never smokers** | | | | | | **Past smokers** | | | | | | **Current smokers** | | | | | | **P** |
| --- | --- | --- | --- | --- | --- | --- | --- | --- | --- | --- | --- | --- | --- | --- | --- | --- | --- | --- | --- | --- |
|  |  | **N_c_** | **N_sub_** | **IRR^a^** | **(95%CI)** | **IRR^b^** | **(95%CI)** | N_c_ | N_sub_ | **IRR^a^** | **(95%CI)** | **IRR^b^** | **(95%CI)** | N_c_ | N_sub_ | **IRR^a^** | **(95%CI)** | **IRR^b^** | **(95%CI)** |  |
| C-592A | CC | 188 | 366 | 1.00 |  | 1.00 |  | 176 | 327 | 1.01 | (0.78-1.31) | 0.95 | (0.73-1.24) | 232 | 397 | 1.16 | (0.91-1.48) | 1.07 | (0.83-1.38) |  |
|  | AC-AA | 94 | 227 | 0.82 | (0.60-1.11) | 0.78 | (0.57-1.06) | 115 | 193 | 1.07 | (0.79-1.44) | 1.01 | (0.74-1.37) | 144 | 264 | 1.08 | (0.82-1.43) | 1.04 | (0.78-1.38) | 0.19 |
| rs3024505 | CC | 200 | 406 | 1.00 |  | 1.00 |  | 190 | 350 | 1.07 | (0.83-1.38) | 1.03 | (0.80-1.34) | 258 | 461 | 1.16 | (0.92-1.46) | 1.11 | (0.87-1.42) |  |
|  | CT-TT | 80 | 191 | 0.89 | (0.65-1.23) | 0.92 | (0.66-1.27) | 98 | 176 | 1.04 | (0.77-1.42) | 1.01 | (0.74-1.38) | 119 | 207 | 1.21 | (0.91-1.62) | 1.16 | (0.86-1.57) | 0.74 |
| C-3737T | CC | 108 | 180 | 1.00 |  | 1.00 |  | 103 | 174 | 0.97 | (0.68-1.38) | 0.94 | (0.65-1.34) | 125 | 215 | 1.05 | (0.75-1.46) | 0.99 | (0.70-1.40) |  |
|  | CT-TT | 170 | 413 | 0.70 | (0.52-0.95) | 0.69 | (0.51-0.94) | 187 | 345 | 0.85 | (0.63-1.16) | 0.80 | (0.59-1.10) | 248 | 445 | 0.93 | (0.69-1.25) | 0.88 | (0.65-1.19) | 0.28 |
| G-1464C | GG | 135 | 325 | 1.00 |  | 1.00 |  | 142 | 279 | 1.18 | (0.88-1.58) | 1.11 | (0.82-1.49) | 177 | 335 | 1.26 | (0.96-1.67) | 1.20 | (0.90-1.61) |  |
|  | GC-CC | 144 | 268 | 1.30 | (0.97-1.74) | 1.29 | (0.96-1.73) | 150 | 243 | 1.37 | (1.02-1.83) | 1.33 | (0.99-1.80) | 198 | 325 | 1.50 | (1.14-1.98) | 1.43 | (1.07-1.89) | 0.86 |
| T-31C | TT | 120 | 276 | 1.00 |  | 1.00 |  | 120 | 231 | 1.18 | (0.86-1.62) | 1.10 | (0.80-1.52) | 149 | 278 | 1.20 | (0.89-1.62) | 1.15 | (0.84-1.56) |  |
|  | TC-CC | 161 | 318 | 1.17 | (0.87-1.57) | 1.15 | (0.86-1.55) | 170 | 292 | 1.22 | (0.91-1.64) | 1.18 | (0.88-1.60) | 226 | 387 | 1.40 | (1.06-1.85) | 1.32 | (0.99-1.75) | 0.90 |
| A-1195G | AA-AG | 266 | 571 | 1,00 |  | 1.00 |  | 278 | 495 | 1,13 | (0.91-1.40) | 1.08 | (0.87-1.34) | 356 | 645 | 1.19 | (0.92-1.40) | 0.92 | (0.92-1.40) |  |
|  | GG | 13 | 22 | 1,23 | (0.59-2.54) | 1.26 | (0.60-2.65) | 14 | 25 | 0,87 | (0.33-2.32) | 0.98 | (0.49-1.95) | 20 | 15 | 2.32 | (0.85-6.28) | 2.33 | (1.13-4.78) | 0.046 |
| G-765C | GG | 208 | 425 | 1.00 |  | 1.00 |  | 221 | 382 | 1.11 | (0.87-1.41) | 1.06 | (0.82-1.35) | 272 | 468 | 1.23 | (0.98-1.54) | 1.17 | (0.92-1.49) |  |
|  | GC-CC | 66 | 161 | 0.89 | (0.63-1.25) | 0.85 | (0.60-1.20) | 68 | 136 | 1.00 | (0.71-1.41) | 0.94 | (0.66-1.33) | 101 | 188 | 1.09 | (0.80-1.47) | 1.02 | (0.74-1.39) | 0.97 |
| T8473C | TT | 125 | 255 | 1.00 |  | 1.00 |  | 132 | 210 | 1.21 | (0.88-1.65) | 1.14 | (0.82-1.58) | 173 | 269 | 1.38 | (1.03-1.86) | 1.29 | (0.95-1.76) |  |
|  | TC-CC | 155 | 335 | 0.98 | (0.73-1.32) | 0.94 | (0.70-1.27) | 150 | 308 | 0.97 | (0.72-1.31) | 0.91 | (0.67-1.24) | 196 | 386 | 1.06 | (0.80-1.41) | 0.98 | (0.73-1.32) | 0.38 |

^a^ Adjusted for sex and age

^b^ In addition, adjusted for smoking status, alcohol, HRT status (women only), BMI, intake of red and processed meat, and dietary fibre

^c^ P p-value for interaction between polymorphisms and smoking status for the adjusted estimates
